# Spatio‐Temporal Patterns of Hybridization in an Alloploid Salamander (Ambystomatidae: *Ambystoma*) and Conservation Implications of Introgression in a Unisexual Vertebrate

**DOI:** 10.1002/ece3.70765

**Published:** 2025-01-26

**Authors:** Alessa V. Laserna, Christopher A. Phillips, Andrew R. Kuhns, Mark A. Davis, Joel B. Corush, Ken N. Paige

**Affiliations:** ^1^ Illinois Natural History Survey, Prairie Research Institute University of Illinois Urbana‐Champaign Champaign Illinois USA; ^2^ School of Integrative Biology University of Illinois Urbana‐Champaign Urbana Illinois USA

**Keywords:** allopolyploid, Ambystoma, conservation, genetic leakage, gynogenesis, hybrid zone, mole salamander, unisexual

## Abstract

Maynard Smith's proposed two‐fold cost of sex states that one of the disadvantages of clonal reproduction is the decreased ability to persist in dynamic ecosystems. However, the long‐term persistence of some clonal alloploid lineages suggests that these lineages may not always be so ephemeral in nature. Understanding the stability of these lineages over time can inform our understanding of the advantages of an asexual mode of life. Here we investigate a gynogenetic allopolyploid triploid, the Silvery Salamander (LJJ—historically referred to as *Ambystoma platineum*), an asexual lineage closely related to *A. barbouri* . However, in our focal populations, neither parental species is present, and another sexual host, *A. texanum* (TT), is a required sperm donor. Gynogenetic reproduction involving LJJ and its host *A. texanum* should result in a clone of the mother (LJJ); however, with the occurrence of kleptogenesis, it often can result in tetraploid hybrids (LJJT). LJJ is considered endangered in this population due to its restricted range coupled with concerns that the cryptic tetraploid (LJJT) could completely replace LJJ. Here we assess the level of LJJ × *A. texanum* hybridization in nine ephemeral wetland populations in east central Illinois. Using species‐specific microsatellite loci, we compared the prevalence of LJJ and LJJT genotypes across localities and years. We find variation across ponds and developmental stages but suggest relative stability over time. Given the considerable amount of environmental degradation and loss, we suggest continued monitoring of this unique segment of biodiversity to ensure its persistence into the future.

## Graphical abstract

Here we investigate a gynogenetic allopolyploid triploid, the Silvery Salamander (LJJ—historically referred to as *Ambystoma platineum*), an asexual lineage. However, in our focal populations, neither parental species is present, and another sexual host, *A. texanum* (TT), is a required sperm donor. We find variation across ponds and developmental stages but suggest relative stability over time.

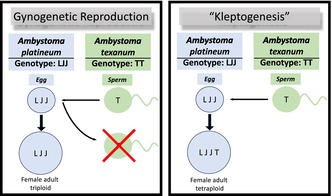

## 1 Introduction

Hybridization yielding allopolyploid, often unisexual vertebrate species, while rare (Neaves and Baumann 2011), is a substantial driver of evolution in natural populations (Bullini 1994; Mallet 2007). Yet in most contexts, these unisexual allopolyploid species can be unstable, and unisexual lineages are expected to be evolutionarily short‐lived (Bi, Bogart, and Fu 2008; Vrijenhoek 1989; Normark, Judson, and Moran 2003) in comparison to sexually reproducing species. This is due, in part, to the lack of recombination, which theoretically renders unisexual species less fit than sexually reproducing organisms (Maynard 1978; Schlupp 2005) and is, in part, a function of the decreased ability to purge deleterious mutations (Muller 1964; Lynch et al. 1993).

**FIGURE 1:**
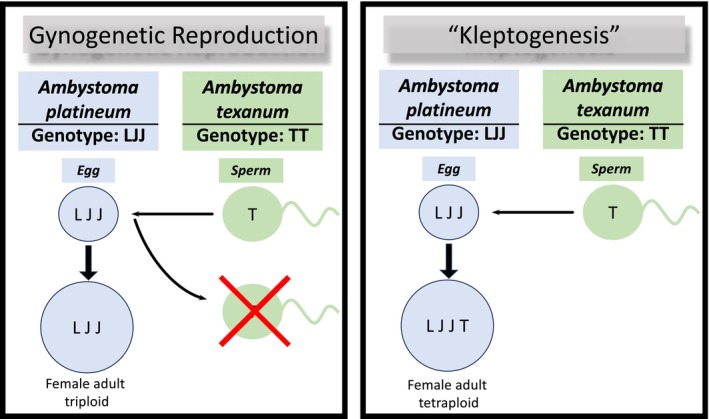
Depiction of gynogenetic reproduction between LJJ (formally known as *Ambystoma platineum* ) and *A. texanum* (Left), in which the *A. texanum* sperm is used to initiate development but the genetic material is discarded. Versus kleptogenesis resulting in fertilization and an increase in ploidy in the female offspring (Right).

Despite these challenges, some unisexual vertebrate mitochondrial lineages are quite old, evolutionarily (Hedges, Bogart, and Maxson 1992; Paquin and Adams 1993). Because many unisexual vertebrates are formed via hybridization of two sexual species (Lamatsch and Stöck 2009; Graf and Pelaz 1989; Lowcock 1989; Kearney, Fujita, and Ridenour 2009), they may possess combination of genotypes not previously observed together within a single lineage (Neaves and Baumann 2011). For example, allopolyploids, individuals who have two or more sets of chromosomes from different species, might display intermediate morphology between the parents as a consequence of co‐dominant genes, creating novel phenotypes (Szymura and Farana 1978) and facilitating physiological and/or behavioral changes that may lead to expanded ranges or increased resilience to environmental change (Greenwald, Denton, and Gibbs 2016; Jakob, Arioli, and Reyer 2010). Additionally, beyond the strict definition of unisexuality, the persistence of ancient unisexual lineages could be due to cryptic introgression among the unisexual and closely related sexual species, acting as a source of introduced genetic diversity with unidirectional exchange of genetic information (Gibbs and Denton 2016). Cryptic introgression would thus result in mitotic recombination, thus circumventing issues associated with Mueller's ratchet and Red Queen dynamics that are typically found in unisexual species (Neiman and Koskella 2009; Denton, Morales, and Gibbs 2018). In unisexual, gynogenetically reproducing organisms, or those in which activation of unreduced ova by sperm is required without genetic contribution from the sperm, introgression has been known to occur (Bogart et al. 2007a; Bogart et al. 2009). Some unisexual *Ambystoma* end up with an additional copy of the genome from the male donor, something not originally considered, given that unisexual *Ambystoma* were only thought to reproduce clonally. Bogart et al. (2007a), Bogart et al. (2007b) termed this mode of reproduction kleptogenesis, arguing that the additional genome may confer an adaptive advantage to their offspring (known as “kleptogens”; Figure 1). Introgression via sympatric sexual hosts in an asexually reproducing organism has been documented across a number of taxa, including many amphibians, reptiles, and fishes (Cuellar and McKinney 1976; Morris and Brandon 1984; Mishina et al. 2021), and is hypothesized to be one of the reasons for the long evolutionary history of some unisexual species, as it facilitates genetic diversity (Verduijn, Van Dijk, and Van Damme 2004; D'Souza et al. 2004) and perhaps even mitotic recombination (Flot et al. 2013). While it is predicted that if introgression is frequent over extended periods of time, unisexuals would become genetically identical to the sexual species (Charney 2012), it is also observed that the percent of kleptogens significantly declines with advancement of developmental stages (Teltser and Greenwald 2015), thus slowing or halting replacement proposed by Charney (2012). However, a significant increase in kleptogen frequencies could result in reproductive effort that is diverted from maintaining the unisex lineage, potentially precipitating population declines.

Given the ostensible rarity of these ancient allopolyploid unisexual species, they represent important segments of biodiversity and may be the focus of conservation initiatives. From this conservation perspective, it is unclear how to deal with the kleptogens in kleptogenetic organisms. Following the strict definition of a “species” based on the biological species concept (Mayr 1942), these individuals are unequivocally hybrids. If an allopolyploid that is experiencing introgression is of conservation concern, is there a responsibility to protect the resulting hybrids along with the putatively “pure” allopolyploid individuals? Furthermore, what does this look like in the case of cryptic hybrids? Most hybrids are not protected under state and/or federal endangered species statutes, and there is persistent debate on whether they should be conserved at all (Allendorff et al. 2001; Stronen and Paquet 2013; Jackiw, Mandil, and Hager 2015).

It is within this context that we investigate these issues in North American unisexual Ambystomids. All unisexual lineages require one of the following sperm donor species: *A. barbouri, A. jeffersonianum, A. laterale, A. texanum*, or *A. tigrinum*, which vary with geography (Bogart and Klemens 2008; Bogart 2019; Wade et al. 2021). The unisexual *Ambystoma* all have the same mitochondrial DNA derived from one parental species, providing evidence that the wide genomic diversity of unisexual *Ambystoma* arose from a hybridization event (Kraus 1989, 1995; Kraus and Miyamoto 1990; Hedges, Bogart, and Maxson 1992). Therefore, we might consider all biotypes of unisexual *Ambystoma* as a continuation of the ongoing speciation process in unisexual *Ambystoma*. In particular, we focus on a peripheral population of polyploid salamanders colloquially known as the “Silvery Salamander,” and historically (see below) recognized as *Ambystoma platineum* , but scientifically recognized and hereafter denoted as LJJ. LJJ, as well as other lineages including LLJ, shares ancestry with Kentucky populations of *A. barbouri* and is thought to be ~5 million years old (Bi and Bogart 2010). LJJ is an all‐female lineage containing two haploid genomes originating from *A. jeffersonianum* (JJ) and one from *A. laterale* (L) and reproduces gynogenetically (McGregor and Uzell 1964). Through gynogenesis, offspring are genetically identical to the mother, maintaining the same ploidy level. The sperm used by the unisexual LJJ usually comes from one of the parental species, *A. jeffersonianum* or *A. laterale* (Lowcock, Licht, and Bogart 1987), but up to three more sperm hosts have been found to be successful donors. These hosts include *A. texanum* , *A. tigrinum,* and *A. barbouri* (designated T, Ti, and B, respectively; Bogart et al. 2009; Bi and Bogart 2010). In some individuals, gynogenesis has been shown to break down, creating tetraploids from the introduction of a haploid sperm into the ova (Morris and Brandon 1984; Spolsky, Phillips, and Uzzell 1992), thus categorizing this system as kleptogenetic (Bogart et al. 2007b). This introgression has been documented in all known sperm donors (as above), creating new cryptic hybrid biotypes (Morris 1985; Bogart 2003). These hybrids maintain two J and one L haploid genomes, but with an additional haploid genome from one of the sperm donors, ploidy is increased. There are also documented, albeit rare, instances of genome replacement (Dawley and Bogart 1989; Spolsky, Phillips, and Uzzell 1992) in which triploid biotypes do not always include one L and two J. Additionally, genome replacement also occurs with LLJ's, which contain two L genomes and one J genome (historically referred to as *A. tremblayi* ) with variation due to fertilization and/or genome replacement (Uzell and Goldblatt 1967; Bogart and Lichts 1986).

We sought to assess the degree and extent of introgression in *A. texanum* ‐dependent LJJ populations in Illinois to evaluate long‐term stability in LJJ and inform the conservation of this unique segment of biodiversity. In our geographic area of study, neither *A. jeffersonianum* nor *A. laterale* are present, and the exclusive sperm host of LJJ is *A. texanum* . And while *A. opacum* co‐occurs with LJJ in our system, their breeding season and courtship behaviors are both temporally asynchronous and ecologically incompatible with LJJ, as *A. opacum* are fall terrestrial breeders and LJJ is a spring aquatic breeder. *A. maculatum* is also present, but larvae fail to hatch from eggs inseminated by *A. maculatum* (Morris and Brandon 1984). No other Ambystomids (including *A. tigrinum* and *A. barbouri*) co‐occur with LJJ in these *A. texanum‐*dependent LJJ sites.

LJJ is of particular interest in Illinois, as it is formally listed as state endangered (as *Ambystoma platineum* ) due to its restricted habitat and at‐risk populations, specifically due to habitat stress caused by fragmentation and disturbance as well as genetic and recruitment‐driven population stress (IDNR 2005; Mankowski 2012; IESPB 2015). Given these concerns, our objectives here were to (1) establish the abundance of tetraploid *A. texanum* ‐dependent LJJ triploid populations in Illinois, (2) investigate survival of triploid LJJ and tetraploid LJJT by quantifying the proportion of adult versus metamorph tetraploids in one breeding season, (3) quantify changes over time in the proportion of triploid: tetraploid genotypes in the populations, and (4) compare water temperature to metamorph ploidy in breeding wetlands to determine if our results are consistent with Bogart, Elisonand, and Licht (1989), which suggest that increased temperatures correspond to increased levels of tetraploids. From a conservation perspective, these data are vital in determining if triploid LJJ is at risk of being lost due to the dynamics of kleptogenesis.

## 2 Methods

### 2.1 Study Sites and Field Sampling

**FIGURE 2:**
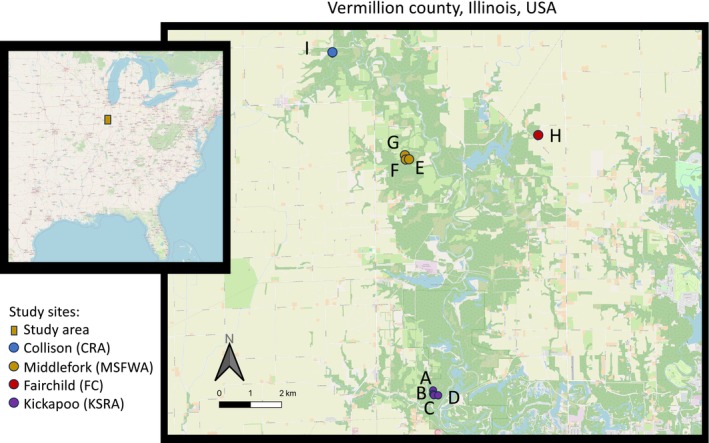
(A) Location of study sites in eastern North America. (B) Map of study sites in Vermillion County in Eastern Illinois, USA. Letters correspond to study wetland listed in Table 1.

We sampled a total of nine ephemeral wetlands from four sites across Vermillion County, IL, USA, known to harbor breeding populations of LJJ and *A. texanum* . This includes four wetlands at Kickapoo State Recreation Area (KSRA), three at Middlefork State Fish and Wildlife Area (MSFWA), one at Fairchild Cemetery (FC), and one at Collison Recreation Area (CRA; Table 1, Figure 2). In addition, we recorded the total number of *A. texanum* and polyploids that were observed, including in past sampling efforts (Table 2).

**TABLE 1:** Adult (2018) and adult and metamorph (2020) LJJ (formally known as *Ambystoma platineum* ; polyploids) and the three locations they were sampled in Vermillion County, IL, and their corresponding pond number and letter.

LJJ ( *A. platineum* ) life stage	Pond location	Pond number	Reference letter	Year		
				2018	2020	2021
Adults	Kickapoo State Recreation Area (KSRA)	66	A		9.5% (42)	
		67	B		11.1% (72)	
		282	C			8.75% (80)
		283	D	14% (100)	31% (100)	11% (100)
	Middle Fork State Fish and Wildlife Area (MSFWA)	75	E		9.1% (33)	
		76	F		36.4% (33)	
		284	G		3.8% (78)	
	Fairchild Cemetery Savanna Nature Preserve (FC)	291	H		2.4% (42)	
	Collison Recreation Area (CRA)	71	I	11% (91)		
Metamorph	Kickapoo State Recreation Area (KSRA)	67	B		8.6% (58)	
		283	D		96.4% (110)	

*Note:* Numbers indicate percent tetraploids with a total number of individual samples in parentheses.

**TABLE 2:** Total number of *Ambystoma texanum* males, *A. texanum* females, and polyploids captured at ponds with pit trap fences at Kickapoo State Recreation Area in 2018, 2020, and 2021.

Pond number	Year sampled	Number of *A. texanum* male	Number of *A. texanum* female	Number of polyploids	*A. texanum* male: polyploids
67 (B)	2018	15	14	152	0.0987
	2020	14	37	64	0.2188
	2021	6	6	113	0.0531
282 (C)	2018	13	13	62	0.2097
	2020	12	5	35	0.3429
	2021	11	11	66	0.1667
283 (D)	2018	47	55	482	0.0975
	2020	42	45	334	0.1257
	2021	3	4	229	0.0131

*Note:* Only a subset of captured individuals were sampled for further analysis (see Table 1). The ratio of *A. texanum* males to polyploids is shown in the last column.

Adult or both adult and metamorphic salamanders were collected using drift fences comprised of 1 m high aluminum flashing, buried 2 cm in the substrate, completely encircling each wetland. A series of 19 L bucket pit traps, buried flush with the ground, were spaced ~5 m apart on both sides of the fence. Buckets on either side of the fence were checked daily when buckets were active (i.e., lids removed to allow capture) in February and March. We opened buckets prior to the first soaking rains of late winter when overnight temperatures were above freezing. Buckets were closed when evening temperatures were predicted to fall below 30°F. At wetlands where drift fences were not established, minnow traps were spaced 1 m apart around the perimeter of the wetland, halfway submerged in water to allow trapped salamanders to breathe. Minnow traps were deployed from February to March and checked for adult salamanders. Samples were collected until ~100 individuals were obtained from each location. Traps were only deployed and checked Monday through Thursdays. If 100 individuals were not collected by mid‐March, traps were removed.

Sampling schedule varied among years due to environmental conditions (e.g., insufficient precipitation, rapidly drying ponds, etc.). During the 2018 breeding season, CRA and one wetland at KSRA (wetland D) were sampled. In 2020, KSRA (A, B, and D), MWFRA (E, F, and G), and Fairchild (H) were sampled for adults. During the summer of 2020, the pitfall trap arrays were used to sample metamorphosing salamanders at KSRA (B and D). In 2021, wetlands at KSRA were sampled for adults (B, D, and G; Table 1). A HOBO temperature logger was deployed in 2020 and 2021 at KSRA B and D. Surface water and substrate temperatures were captured hourly from February to April to assess whether there was a relationship between ploidy levels and temperature as in Bogart, Elisonand, and Licht (1989).

### 2.2 Genotyping

Tissue samples were genotyped from representative samples of individuals collected in 2018, 2020, and 2021. Adult salamanders were visually identified, as unisexual polyploids are morphologically/phenotypically distinct from *A. texanum* , and either an approximate 5 mm section of the distal tail, or half of a hind‐limb toe was clipped using sterile scissors. Tissues were immediately preserved in 100% ethanol, transported back to the laboratory on ice, and stored at ‐20 C until extraction. DNA was extracted via the Qiagen dNeasy Blood and Tissue Kit (Qiagen Inc., Valencia, CA) using standard protocols.

Ploidy was determined for each individual by amplifying a microsatellite locus only present in *A. texanum* . For adult and metamorph samples, the microsatellite Atex133 of Williams and DeWoody (2003; Accession AY362350) was amplified in 50 μL reactions containing 50 ng of DNA, 1 U of Taq DNA Polymerase, 1.5 mM MgCl_2_, 0.25 mM dNTP (all from New England Biolabs), and 0.5 μM of each primer. All DNA samples were standardized to 50 ng/uL using deionized water. Thermocycler conditions involved an initial denaturation step at 94°C for 2 min, 34 cycles of denaturation at 94°C for 30 s, followed by an annealing temperature at 57.5°C for 30 s, and an extension at 68°C for 30 s. The final extension was 68°C for 5 min. PCR products were separated in a 1% agarose gel, and samples that displayed an approximate 500 bp band of the AtTex133 primer were designated as LJJT (Figure S1).

In the case of metamorph samples, a second primer was used to genetically confirm the LJJ identity, as many species in these stages are difficult to identify visually. Because *A. jeffersonianum* is not found in our study sites (Phillips, Crawford, and Kuhns 2022), microsatellites from Julian, King, and Savage (2003) found in *A. jeffersonianum* were screened, and aJeD294 was selected. Since *A. jeffersonianum* is one of LJJ's parental species, individuals displaying a band for aJeD294 and not for Atex133 were determined to be LJJ, and individuals with bands for both aJeD294 and aTex133 were determined to be LJJT. Amplification using aJeD294 was completed in a 7.5 μL reaction containing 3.75 μL of Qiagen Taq PCR Master Mix, 1.7 μL H_2_O, 0.4 μL of both forward and reverse aJeD294 primer at 10 nM, and 1.25 μL of DNA. Thermocycler conditions involved an initial denaturation step at 94°C for 2 min, 35 cycles of denaturation at 94°C for 45 s, followed by an annealing temperature at 58°C for 45 s, and an extension at 72°C for 90 s. The final extension was 72°C for 5 min. PCR products were separated in a 1%–2% agarose gel stained with ethidium bromide and visualized and photographed under UV light for subsequent scoring. Negative control reactions with no DNA template and positive controls with parental *A. jeffersonianum* , *A. laterale* , *and A. texanum* tissues were used in each PCR (see Figure S1 for an example panel of all samples used). Because of the species specificity for both aJeD294 and aTex133 microsatellites (Figure S1), sequencing and subsequent fragment analysis were unnecessary. One concern of this methodology is distinguishing between LJJT and the occurrence of triploids that potentially have had genome replacement (i.e., LJT) or higher levels of ploidy (LJJTT). Bogart (2019) reported genome replacement in 5 of 988 (0.5%) of individuals (LLJ females producing LJJ offspring). Spolsky, Phillips, and Uzzell (1992) found no LJT out of 32 individuals. Given the low probability of LJT occurrence in our sample size, coupled with the additional required methodologies of detecting triploids versus tetraploids, we were unable to detect LJT individuals.

In addition to the genotypic data described above, data from previous studies that also sampled individuals in KSRA‐D were compiled. Ploidy data based on erythrocyte area from 16 individuals collected in 1980 (Morris and Brandon 1984) and allozyme data collected from 60 individuals from 1990 to 1992 (Spolsky, Phillips, and Uzzell 1992) were compared to ploidy data collected here to identify temporal patterns. Because samples were collected from the same location, issues pertaining to different sperm‐donor species and other *Ambystoma* interactions were of no concern.

### 2.3 Data Analyses

A two tailed Grubb's outlier test was used to determine if there were any outliers in the tetraploidy levels in adults during 2020 and for the temporal tetraploidy data at KSRA‐D using the “grubbs.Test” function in the “outlier” package in R (R core team 2021). Differences in average water temperatures among ponds (KSRA‐A and KSRA‐B; 2020) and across years (2018 and 2020; KSRA‐A) were determined using a two‐tailed *t*‐test.

## 3 Results

We found tetraploids (LJJT) in all nine *A. texanum* ‐dependent LJJ wetlands sampled during this study. There were neither sampled wetlands nor years without tetraploids. Adult tetraploid abundance varied from 2.4% (pond: FC‐H, year: 2020) to 36.4% (pond: MSFWA‐F, year: 2020), with a mean of 13.5% across all years and ponds (Table 1). Values representing both the upper and lower portions of the range were all found within the sites consisting of multiple ponds and sampled over multiple years (KSRA and MSFWA). While we found there to be variation across the seven ponds sampled in 2020, no value was considered an outlier (*G* = 1.59402, *U* = 0.50594, *p* = 0.547; Table 1). The proportion of adult male *A. texanum* to adult polyploids also varied across ponds and years, ranging from 0.01 to 0.34 (Table 2).

At KSRA‐D, we compared our data with published historical data (Morris and Brandon 1984; Spolsky, Phillips, and Uzzell 1992) from the same wetland in an attempt to reveal longer‐term trends. Here we reveal transient dynamics in the proportion of triploid and tetraploid individuals between 1980 and 2021 (1980 = 14%, 1990 = 7%, 1991 = 28%, 1992 = 13%, 2018 = 14%, 2020 = 31%, 2021 = 11%). Neither the smallest (7%, 1990) nor the largest (31%, 2020) values were found to be outliers (*G* = 1.57004, *U* = 0.52069, *p* = 0.5932).

For the two ponds sampled for metamorphs, tetraploid abundance was 8.6% (pond: KSRA‐B, year 2020) and 96.4% (pond: KSRA‐D, year 2020; Table 1). In 2020, the abundance of tetraploids was over 3 times greater for metamorphs than adults at KSRA‐D, while the abundance of tetraploids was similar in adults versus metamorphs at KSRA‐B (Table 1).

In 2018, the average water temperature at KSRA‐D was 4.8°C ± 4.2°C (see Table S1 for data). In 2020, the average temperature at KSRA‐B was 5.8°C ± 3.7 and 6.6°C ± 4.2 at KSRA‐D. The average water temperature between KSRA‐D and KSRA‐B was significantly different (*p* = 0.004), and water temperatures between 2018 and 2020 at KSRA‐D were significantly different (*p* = 0.0001).

## 4 Discussion

Our results show that the allopolyploid/unisexual triploid ambystomatid salamander (LJJ) and its diploid host ( *A. texanum* ) produce tetraploid (LJJT) individuals via kleptogenesis among wetlands across eastern Illinois, albeit at varying proportions and with no apparent spatio‐temporal pattern. Though tetraploid presence is widespread, the proportion of tetraploid LJJT to triploid LJJ is consistent with previously published data in populations outside the range of this study. For example, within the LJJ system, Lowcock (1994) report tetraploid values of 12.3%, 2.6%, 0%, 0%, 11.1%, and 0% across six different central Ontario localities. Lowcock, Griffith, and Murphy (1991) reported slight fluctuation from a single location between 1988 and 1991; 15.35%, 20.73%, 21.30%, and 19.69%. Similar values are also reported in other gynogenic systems that also, on occasions, incorporate male genomes. For example, within the gynogenetic *Phoxinus eos‐neogaeus* clonal fishes, 22%–33% of individuals across four natural populations showed incorporation of a third set of chromosomes from males of the parental species (Doeringsfeld et al. 2004). However, there may not be a single optimal percentage necessary for the maintenance of these asexual lineages, but rather balancing selection for and against the maintenance of sexual individuals based on population‐specific conditions (Schlupp 2005; Mee and Otto 2010). Our compilation of longitudinal ploidy data over 41 years at a single wetland (KSRA‐D) showed no differences between average levels of ploidy measured between 1980 and 2021. Tetraploidy estimates at KSRA‐D indicate that the proportion of LJJT adults fluctuates around a mean of 17%. This balanced kleptogenesis of sexual genomes into the unisexual lineage may arguably be a defining characteristic of this complex (Denton, Morales, and Gibbs 2018).

In line with Teltser and Greenwald (2015), we revealed evidence of a developmental shift in ploidy. Early developmental stages (i.e., metamorphs) exhibited a greater percentage of tetraploids compared to adults. Of adults sampled at KSRA‐D during the breeding season in 2020, 31% were LJJT, whereas 96.4% of the metamorphs were LJJT. No larvae were sampled at KSRA‐D, but our results at this wetland are consistent with those of Teltser and Greenwald (2015), in which they observed a gradual decline in ploidy levels as developmental stages progressed. This was not the case at KSRA‐B, where the percent of LJJT metamorphs (8.6%) was similar to the percent of LJJT adults (11.1%). Obtaining data to support the hypothesis of differential mortality between triploids and LJJT posthatching would require ploidy determination between each developmental stage, starting with eggs. Also in line with Teltser and Greenwald (2015), as well as Bogart, Elisonand, and Licht (1989), we found higher proportion of tetraploids in water with higher average temperatures, however our observed water temperatures were only marginally higher (~1°C) and, due to the small sample size, no statistical significance can be attributed to this trend.

KSRA‐D has the largest number of unisexual LJJ and LJJT metamorphs emerging from it compared to nearby wetlands. In 2020, a combination of 266 LJJ and LJJT metamorphs emerged from KSRA‐D, whereas only 101 metamorphs emerged from KSRA‐B. This could be concerning for the population of LJJ at KSRA as the largest and most productive wetland that was sampled (D) predominantly produced LJJT (106 out of 110 individuals sampled). The worry regarding LJJ hybridization has been that a large majority of recruits are in fact LJJT. This concern has been substantiated, as *A. texanum's* spermatophore deposits have been shown to be larger for LJJT than for LJJ, indicative of a breeding preference for females containing an *A. texanum* genome (Phillips et al. 1997). This might explain the greater number of tetraploid metamorphs produced relative to triploids. Additionally, production of LJJT could arise via two different pathways, either through normal gynogenesis of a tetraploid or through the fertilization of an unreduced triploid egg, increasing the probability of offspring having increased ploidy (Morris and Brandon 1984; Spolsky, Phillips, and Uzzell 1992). Although 96.4% of tetraploids at KSRA‐D is concerning for the population of LJJ at KSRA, as that implies that most of the reproductive efforts of LJJ went to individuals with low fitness, tetraploid abundance was much lower at KSRA‐B and even slightly lower in metamorphs compared to adults at this wetland. Since tetraploid percentages fluctuate annually, it is likely the high number of tetraploids at KSRA‐D are a result of annual fluctuations in the rate of kleptogenesis. Additional years of metamorph ploidy data are necessary to assess the severity of hybridization at KSRA‐D. Temporal variation within the year may also be an important contributing factor. Because our samples were opportunistically collected based on weather conditions, we might not have captured variation that occurs over the course of a full year.

Wetlands within the same sites experienced ploidy variation even when ponds were located proximal to one another. Ambystomatids typically exhibit high natal philopatry, returning to the same pond to breed every year (Schoop 1965; Douglas and Monroe 1981; Raymond and Hardy 1990). Thus, higher percentages of tetraploidy at a wetland could give insight into potential causes for increased abundance of tetraploidy in comparison to wetlands at the same site that have lower abundance. For example, wetlands KSRA‐D and ‐B exhibited differential tetraploidy abundance for adults, despite their relatively close proximity to one another. Tetraploidy abundance for adults at KSRA‐D was 31% in 2020, while only 11% at KSRA‐B in the same year, an approximately 3‐fold difference.

There has been a longstanding debate on whether hybrids should be conserved along with species of conservation concern (Rhymer and Simberloff 1996; Allendorff et al. 2001; Fitzpatrick et al. 2015). Historically, the majority of conservation policy has disfavored the conservation of hybrid species (Mayr 1942; Agapow et al. 2004; but see O'Brien and Mayr 1991; Whitham, Morrow, and Potts 1991), often viewing hybrids as unfavorable to genetically pure, nonambiguous species from which the hybrid was derived. Nevertheless, the state of Illinois has listed LJJ ( *A. platineum* ) as a threatened species, requiring the protection of *A. texanum* due to the gynogenetic behavior observed in LJJ; however, the levels of kleptogenesis are not considered.

Of particular conservation concern are anthropogenically driven changes in water temperature, which are expected to have an effect on the abundance of tetraploid hybrids (Bogart et al. 2007a; Teltser and Greenwald 2015). Although wetland creation could be extremely beneficial to the amphibian communities more broadly (Ruhí et al. 2012, but also see Bellio, Kingsford, and Kotagama 2009), this study should serve as a cautionary tale for creating wetlands with the specific needs of the species inhabiting them in mind. Wetlands created too shallow or with a lack of shade could result in different temperature profiles compared to similar natural habitats. In the case of LJJ, improper restoration efforts may shift the frequency of kleptogenesis.

On the other hand, conserving LJJT along with LJJ could prove helpful in the persistence of the lineage as recombination may provide novel genotypes. Introgressive hybridization could increase individual fitness and expand the range of suitable ecological conditions in comparison to the parental lineages (Paquin and Adams 1993; Neaves and Baumann 2011; Gao et al. 2009; Seehausen 2004; Burger et al. 2024). For example, triploid unisexual salamanders (LLJ) have been found to be more prevalent in habitats with increased anthropogenic disturbance, higher elevation and lower temperatures; suggesting triploids have a wider geographical range in comparison to the bi‐parental species, potentially due to the additional genome in triploids (Greenwald, Denton, and Gibbs 2016). The additional genome could also facilitate novel physiological, morphological and/or behavioral adaptations that increase hybrid fitness in novel environments (Jakob, Arioli, and Reyer 2010). Keller and Gerhardt (2001) demonstrated that the doubling of genomes resulting from autopolyploidy (diploid to tetraploid) in *Hyla versicolor* changed their mating call to allow *H. versicolor* females to identify conspecifics and avoid hybridization with the parental species, *Hyla chrysoscelis* . The outcome of both of these examples would result in improved fitness, either by increasing the tolerance of ecological conditions or adding adaptations that would help them remain in or disperse into new territory. Spontaneous heterosis (“hybrid vigor”) could also explain a fitness increase in the hybrids (Lippmann and Zamir 2007) through increased heterozygosity or recombination. From an evolutionary perspective, hybridity has been a natural part of species' adaptation and speciation, leading to increased species richness (Vila, Weber, and D'Antonio 2000; Seehausen 2004).

It is important to consider whether tetraploid hybrids play a negative role in a population's persistence. Future studies should focus on genome‐scale sequencing to quantify the level of recombination between the unisexual hybrid and paternal sexual host. Future ploidy sampling should be done with a wide range of developmental stages, particularly in ponds with above‐average levels of tetraploids. Additionally, it is important that we delve into potential causes for the increased fertilization rates that lead to ploidy increase in *A. texanum‐*dependent LJJ populations to learn whether it is anthropogenically caused by factors such as climate change altering water temperature, a potential threat based on Teltser and Greenwald (2015), as well as Bogart, Elisonand, and Licht (1989), or other factors as yet unaccounted for. This study shows that even though hybrid LJJTs were found at all *A. texanum‐*dependent LJJ ponds sampled, they do not seem to pose a threat to LJJ, and time and resources should be focused on the conservation of the complex as a whole. This should include *A. texanum*, as persistence of LJJ depends on the sexual host.

## Author Contributions

**Alessa V. Laserna:** conceptualization (equal), formal analysis (equal), investigation (equal), writing – original draft (lead), writing – review and editing (equal). **Christopher A. Phillips:** conceptualization (equal), formal analysis (equal), investigation (equal), writing – original draft (supporting), writing – review and editing (equal). **Andrew R. Kuhns:** conceptualization (equal), formal analysis (equal), funding acquisition (equal), investigation (equal), writing – original draft (supporting), writing – review and editing (equal). **Mark A. Davis:** conceptualization (equal), formal analysis (equal), investigation (equal), writing – original draft (supporting), writing – review and editing (equal). **Joel B. Corush:** conceptualization (equal), formal analysis (equal), investigation (equal), writing – original draft (supporting), writing – review and editing (equal). **Ken N. Paige:** conceptualization (equal), formal analysis (equal), funding acquisition (equal), investigation (equal), writing – original draft (supporting), writing – review and editing (equal).

## Conflicts of Interest

The authors declare no conflicts of interest.

## Supporting information


Figure S1.

Table S1.

## Data Availability

Datasets underlying this work are available in Table 1 within this article and Figure S1 and Table S1.
