# Supplementary material for: Spatio‐Temporal Patterns of Hybridization in an Alloploid Salamander (Ambystomatidae: Ambystoma) and Conservation Implications of Introgression in a Unisexual Vertebrate
Source: Ecol Evol. 2025 Jan 26;15(1):e70765. doi: 10.1002/ece3.70765 (PMC11770251; doi:10.1002/ece3.70765)
Supplement: Supplementary file 1 — Figure S1. [file ECE3-15-e70765-s001.docx]

Supporting Information (Table S1, Figure S1):

Table S1. Surface water temperature (in elsius) taken during the breeding season in 2020 at Kickapoo State Recreation Area at ponds 283 and 67. Temperatures are included from the first breeding migration into the pond to the end of the breeding season when *A. platineum* stopped moving into the ponds.  See attached file: Temperature_Data.xlsx


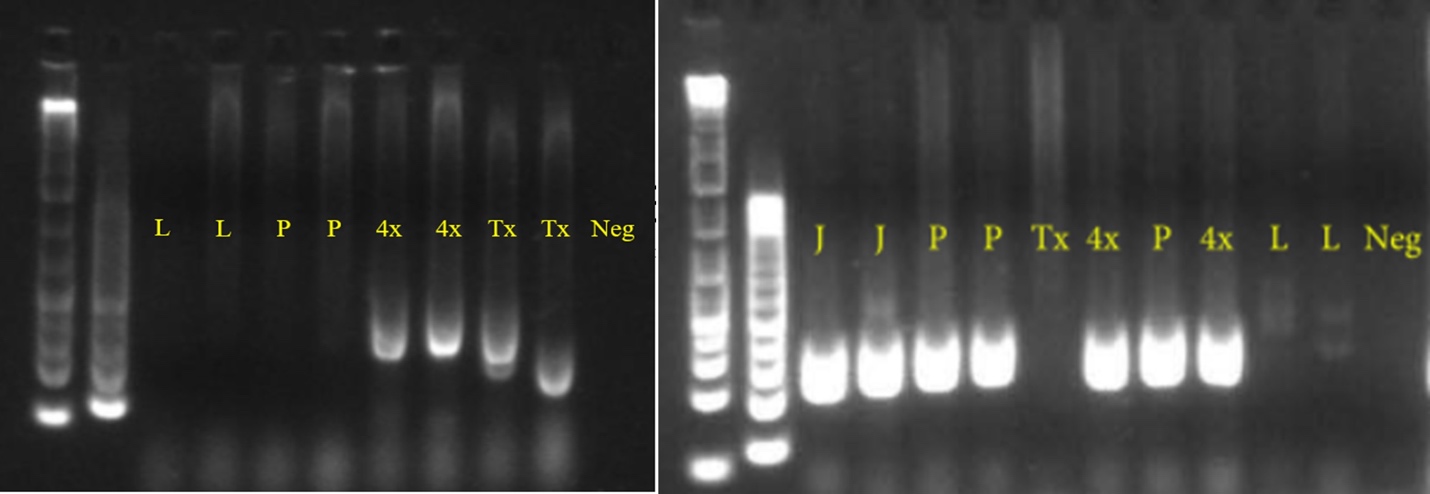

Figure S1. Gel electrophoresis band pattern for primers aTex133 (left) and aJeD294 (right) using *Ambystoma jeffersonianum* (J), *A. laterale* (L), *A. platineum* (P), *A. texanum* (T) and LJJT (4x). 
